# A Comparative Study of Geoelectric Signals Possibly Associated with the Occurrence of Two *M_s_* > 7 EQs in the South Pacific Coast of Mexico

**DOI:** 10.3390/e21121225

**Published:** 2019-12-15

**Authors:** Lev Guzmán-Vargas, Carlos Carrizales-Velazquez, Israel Reyes-Ramírez, Jorge Fonseca-Campos, Arturo de la Rosa-Galindo, Víctor O. Quintana-Moreno, José Antonio Peralta, Fernando Angulo-Brown

**Affiliations:** 1 Unidad Profesional Interdisciplinaria en Ingeniería y Tecnología Avanzada, Instituto Politécnico Nacional, Ciudad de México 07340, Mexico; 2 Instituto Tecnológico de Pinotepa, Santiago Pinotepa Nacional C.P. 71600, Oaxaca, Mexico; 3 Universidad Tecnológica de la Costa Grande de Guerrero, Petatlán 40830, Guerrero, Mexico; 4 Departamento de Física, Instituto Politécnico Nacional-Escuela Superior de Física y Matemáticas, Ciudad de México 07738, Mexico

**Keywords:** self-potential, ULF-range, correlations, main shock

## Abstract

During past decades, several studies have suggested the existence of possible seismic electric precursors associated with earthquakes of magnitude M>7. However, additional analyses are needed to have more reliable evidence of pattern behavior prior to the occurrence of a big event. In this article we report analyses of self-potential ΔV records during approximately two years in three electro-seismic stations in Mexico located at Acapulco, Guerrero; Petatlán, Guerrero and Pinotepa Nacional, Oaxaca. On 18 April 2014 an $M_{s}7.2$ earthquake occurred near our Petatlán station. Our study shows two notable anomalies observed in the behavior of the Fourier power spectrum of ΔV for ultra low frequency ULF-range, and the transition of the $\alpha_{l}$-exponent of the detrended fluctuation analysis of the ΔV time series from uncorrelated to correlated signals. These anomalies lasted approximately three and a half months before the main shock. We compare this electric pattern with another electric signal we reported associated with an $M_{s}7.4$ that occurred on 14 September 1995 at Guerrero state, Mexico. Our characterization of the anomalies observed in both signals point out similar features that enrich our knowledge about precursory phenomena linked to the occurrence of earthquakes of magnitude M>7.

## 1. Introduction

Nowadays it is increasingly accepted that Earth’s crust is a self-organized critical system [1,2,3]. This fact is not favorable for seismic prediction. The concept of self-organized criticality (SOC) was coined by Bak et al. [4,5,6] to describe the collective behavior of complex macroscopic open systems constituted by many objects without a predominant scale; that is, systems with fractal geometry that only reach metastable states. Evidently, Earth’s crust has these kinds of properties and seismicity is a dynamical expression of its SOC nature. The SOC systems produce avalanches of a great variety of sizes, only limited by the finite size of the system itself. That is, given a certain avalanche, the size and the occurrence time of the next avalanche is unpredictable. If Earth’s crust is actually a SOC system, then the earthquakes are unpredictable. However, there are other SOC processes like rain [7,8,9], which in spite of being long-term unpredictable in the SOC sense it can be short-term predictable when the signs of imminence of a huge storm are identified. The appearance of these signs usually is present only some minutes before the main event is unleashed. In the case of storms the precursory signs are visible, but in the case of big earthquakes the possible precursory signs in general are hidden underground. Thus, one can attempt an analog approach to the short-term storm prediction for the case of big earthquakes. For several decades, many research groups around the world have been interested in the identification of the mentioned precursory phenomena of earthquakes [10,11,12,13,14,15,16,17]. One of the more studied precursory phenomena has been the measurement of geoelectric activity in very active seismic zones, like Greece [11,12,13] and Japan [14,15,17]. Varotsos et al. [17] published a recent work where they found emergence phenomena almost three months before the M9 giant Tohoku earthquake on 11 March 2011. The fact that the Tohoku earthquake (EQ) occurred after the emergence of almost random behavior did not come as a surprise since it is strikingly reminiscent of similar findings in other complex time series. For example, in the case of electrocardiograms, the long-range temporal correlations characterize the healthy heart rate variability breakdown for individuals at high risk of sudden cardiac death and this is often accompanied by the emergence of uncorrelated randomness, e.g., see [18]. Moreover, there were found according to the seismic electric signals (SES) activities; the SES observation marks when the system enters a critical stage and infinitely long-range correlations are present [19].

Our group, since 1993 have intermittently measured the ground electric potential (the self electric potential) in several sites along the Mexican Pacific coast, near the Middle North-American Trench, which is the border between the Cocos and North-American tectonic plates. In some previous articles, we have reported more detailed descriptions of that region and some studies of possible precursory geoelectric phenomena associated to several earthquakes with magnitude Ms≥6 [16,20,21,22,23]. Typically, the electric potential time series generated in our geoelectrical stations [16,24] are very noisy; however, after the application of some methods stemming from nonlinear dynamics and statistical physics one can extract hidden information. Among the used methods are: The Fourier power spectrum [16], the Higuchi fractal dimension [22,25], the detrended fluctuation analysis (DFA) [24,26] and the multifractal spectra [20]. In general, we have identified a kind of pattern consisting of a long period in the order of many months or years where only white noise (WN) is observed. After the WN period, suddenly color noise (CN) appears (brown or pink) and is present for some few months [24]. After the CN period, a big earthquake ($M_{s}=7.4$ and $M_{s}=7.2$, see Section 4 of the present article) occurs. A short time after the occurrence of these EQs, the uncorrelated WN signal is recovered. Here we present two noticeable anomalies in the behavior of the Fourier power spectrum of self-potential signals from a station in Petatlán, Guerrero, México together with an alteration of the correlation α-exponent from uncorrelated to correlated dynamics. We identified that these anomalies lasted approximately three and a half months before the main shock. In addition, we compare these anomalies with a previous study of an $M_{s}7.4$ that occurred on 14 September 1995 in Guerrero state, Mexico. Our results about the similarities of both anomalies reinforce the idea of the presence of precursory patterns before the occurrence of a big earthquake. The present article is organized as follows: In Section 2 we present a brief description of our stations, and details of the measurement of ΔV data in real time; in Section 3 we explain the methods; finally in Section 4, we present some remarks and conclusions.

## 2. The Stations

**Figure 1:**
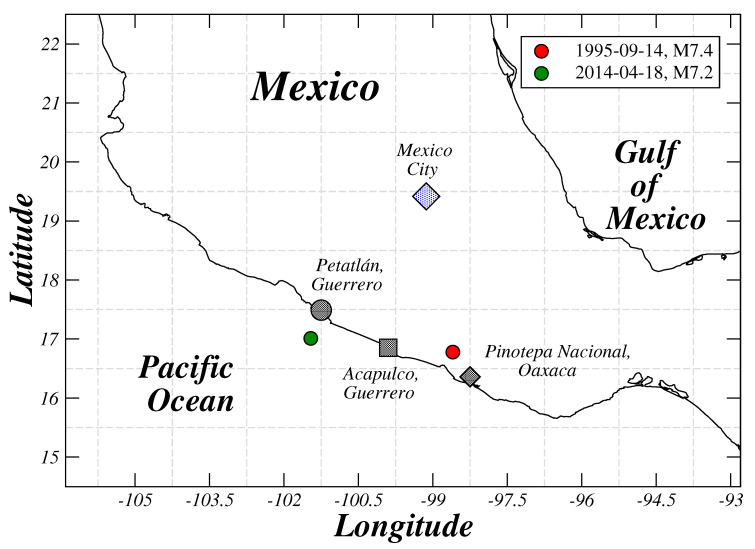
Map of locations of Acapulco, Petatlán and Pinotepa stations. The locations of the epicenters of the studied earthquakes are also shown.

**Figure 2:**
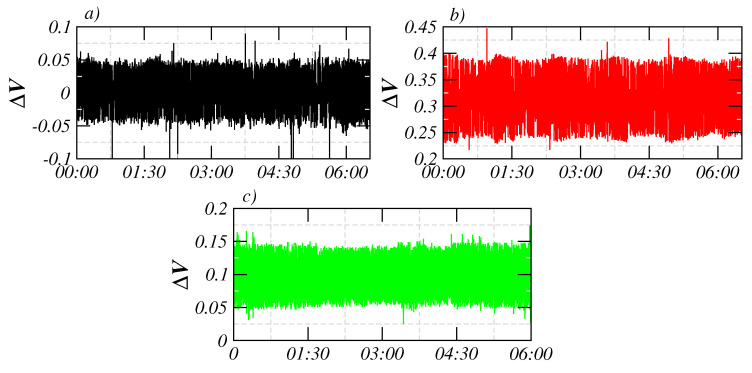
Representative segments of the signals from (**a**) Petatlán, (**b**) Pinotepa, and (**c**) Acapulco stations. These segments correspond to the first six hours of October (2013 for Petatlán and Pinotepa stations, and 1994 for Acapulco station).

The electric self-potential difference ΔV between four pairs of electrodes was collected in stations located on the South Pacific Coast of Mexico. The electrodes were buried 1 m into the ground. Two pairs of electrodes were arranged in a north–south (N-S) direction and the other pairs in an east–west (E-W) direction. The distance between electrodes was 20 m for the first pair and 40 m for the second one. Our electrode layout to measure ground self-potentials is similar to the one from Varotsos in Greece [11], which is recognized as the VAN methodology [27]. We report results of measurements from three stations, the Acapulco station (Aca) (16.85 N, 99.9 W), which was active from June 1994 to December 1996 [24]; the Pinotepa station (Pin) (16.36 N, 98.25 W) active from November 2012 to May 2014 and the Petatlán station (Pet) (17.49 N, 101.25 W) active from January 2013 to May 2014 (see Figure 1 and Figure 2). Four signals were simultaneously recorded at each station (N-S and E-W short and large channels). The sampling rate was $f_{s}=0.5$ s${}^{-1}$ for all stations. A more detailed description of the geoelectrical station can be seen in [16].

## 3. Methods

### 3.1. Spectral Power and Frequency-Band Analyses

Fourier transform is used to decompose a time series $\left\{x_{n}\right\}$ in a summation of sinusoidal waves with well-determined frequency [28]. In the discrete domain, the transformation is given by:

(1) $$X_{k}=\sum_{n=1}^{N}x_{n}\exp^{-i2\pi kn/N},\;k=1,2,3,\ldots ,\frac{N}{2}.$$

where the Fourier coefficients $X_{k}$ are associated with frequencies $f_{k}=k/N$. In the frequency domain, a measure of the energy distribution of a signal as a function of frequency is $|X_{k}{|}^{2}$. As *N* approaches infinity, the total energy diverges and also approaches infinity. In consequence, it is standard practice to convert the energy to power [29]. The power-spectral density function of $x_{n}$ is defined as

(2) $$S_{k}=\lim_{N\to \infty }\frac{|X_{k}{|}^{2}}{N/2}.$$

The distribution of $S_{k}$ is informative of how the frequencies are contributing to the spectral power. For instance, a well-defined peak in $S_{k}$ indicates that the frequency interval for which the peak occurs is dominating the oscillations. In many real situations, $S_{k}$ exhibits non-well-defined maxima with no clear increasing or decreasing tendency along the frequency interval. A better quantification of the contribution of frequency intervals can be obtained if the frequency interval is divided into bands, allowing a more informative way of evaluating frequency contribution to the total power of the signal. This approach has been used in previous studies [16] to quantify power-frequency dependence in geoelectrical signals as a possible seismic precursor.

In Reference [30] Kantelhardt et al. used a method to analyze complex signals by means of a segmentation of the frequency spectrum in frequency intervals. Yépez et al. [16] also used a segmentation method to analyse geoelectrical signals in 1995. Briefly, the signal under study is decomposed into frequency components employing the Fourier filtering technique described above. We analyze 8 bands for which the entire frequency interval is partitioned into 8 equal-sized bands with intervals in increasing order ($\left\{\Delta f_{j}\right\}$, j=1,2,..,8). For the purpose of our study, the specific intervals are: $\Delta f_{1}=$ [0 Hz–0.015 Hz], $\Delta f_{2}$ = [0.015 Hz–0.03 Hz], $\Delta f_{3}$ = [0.03 Hz–0.045 Hz], $\Delta f_{4}$ = [0.045 Hz–0.06 Hz], $\Delta f_{5}$ = [0.06 Hz–0.075 Hz], $\Delta f_{6}$ = [0.075 Hz–0.09 Hz], $\Delta f_{7}$ = [0.09 Hz–0.105 Hz] and $\Delta f_{8}$ = [0.105 Hz–0.12 Hz]. For each band $\Delta f_{j}$, all Fourier coefficients, except those of the desired band, are set to zero, and the inverse Fourier transform is applied to get the filtered signal back into the time domain ($x_{n}^{\left(j\right)}$), where the frequencies corresponding to the *j*-th band are present. We also use the Hilbert transform to calculate the instantaneous amplitudes (IA) and the instantaneous frequencies (IF) of the filtered signal. The imaginary component ${\tilde{x}}_{n}^{\left(j\right)}$ is used to get:

(3) $$x_{n}^{\left(j\right)}+i{\tilde{x}}_{n}^{\left(j\right)}=A_{n}^{\left(j\right)}\exp\left(i\phi_{n}^{\left(j\right)}\right),$$

where IA are given by $A_{n}^{\left(j\right)}$ and IF are calculated (by using the instantaneous phases $\phi_{n}^{\left(j\right)}$) as:

(4) $$f_{n}^{\left(j\right)}=\left|\phi_{n}^{\left(j\right)}-\phi_{n-1}^{\left(j\right)}\right|.$$

### 3.2. Detrended Fluctuation Analysis

Detrended fluctuation analysis (DFA) is a useful technique to analyze long-range correlation in a time series [24,31,32,33]. First, the signal $\left\{X_{i}\right\}$ is integrated; the resulting series ($Y_{i}$) is divided into windows of size *s* and, for each window, a straight line is fitted to the points ($Y_{n}$). Next, the root-mean-square fluctuation of the detrended sequence within each window is computed:

(5) $$F\left(s\right)=\sqrt{\frac{1}{N_{s}}\sum_{i=1}^{N_{s}}{\left[Y_{i}-Y_{n}\right]}^{2}}.$$

If the original time series is self-similar, F(s) will follow a power-law behavior of the form $F\left(s\right)∼s^{\alpha }$, where the scaling exponent α characterizes the correlations in the series. It is well known that α=0.5 corresponds to uncorrelated fluctuations, α=1 refers to long-range correlated signals (1/f-noise or pink noise), and α=1.5 corresponds to a Brownian motion where the increments are totally uncorrelated [24].

## 4. Results

As we mentioned in Section 2, four signals were simultaneously recorded at each station, corresponding to the N-S and E-W channels. In what follows, we focused on N-S short (distance between electrodes of 20 m) signals since similar results were obtained for the rest of the channels [24].

### 4.1. Spectral Analysis

**Figure 3:**
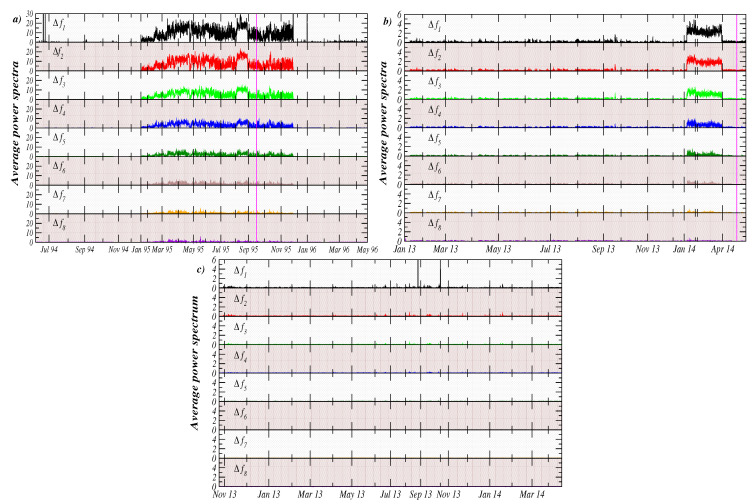
Average power spectrum values as a function of time for eight frequency bands from three stations in the South Pacific Coast in México. (**a**) Acapulco data for the period June 1994 to May 1996. The vertical line indicates the occurrence of the $M_{s}7.4$ EQ, 14 September 1995. (**b**) Petatlán data for the period January 2013 to May 2014. The vertical line indicates the $M_{s}7.2$ EQ, 18 April 2014. (**c**) Pinotepa Nacional data for the period November 2013 to May 2014.

Figure 3a shows the average power spectrum density (APSD) value for the Acapulco station as a function of time. The average values are presented for each frequency band (see ’Methods’ for details). We observe that from the beginning of July 1994 until November 1994, the APSD values are very small (close to zero) in all frequency bands. After this period, the APSD started to increase, especially for bands corresponding to very low frequencies. This behavior started in January 1995 and lasted until the end of December 1995. After the beginning of 1996, the contributions of the frequency bands to the average power returned to their baseline, which is close to zero, except for some small peaks observed during 1996 in low frequency bands (see Figure 3a). Our quantitative analysis based on power density indicates that there was a clear transition pattern from a flat spectrum (uncorrelated fluctuations) to a right-skewed spectrum, where major contributions to the power are given by low-frequency intervals, and end again with flat spectrum behavior.

The same analysis was performed in the case of the Petatlán station.Figure 3b presents the results for the period January 2013 until the end of May 2014. For this case, we observe that, for the period January 2013 to December 2013, the values of APSD are quite low (close to zero), which resemble the characteristic behavior of uncorrelated fluctuations. Notably, an abrupt increase of the APSD is observed at the beginning of January 2014, remaining at high values until the beginning of April 2014 and then APSD values returned to their baseline values associated to random fluctuations. It is very interesting that an $M_{s}7.2$ EQ occurred on 18 April 2014 at ∼57 km from this station. Remarkably, the geoelectric anomaly in the APSD ended four days before the big earthquake (see Figure 3b). In general terms, the sequence of changes in the APSD was observed at the Petatlán station; the uncorrelated signal-correlated signal-EQ-uncorrelated signal is similar to the sequence observed almost 25 years ago at the Acapulco station (see Figure 3a).

As a control, we also repeated our calculations of APSD to data from a third station located in Pinotepa, Oaxaca, Mexico. The reason for including this station in this study is that during the period of observation (November 2013 to March 2014), no significant EQ-activity in the vicinity of the station was reported. As shown in Figure 3c, APSDs in all frequency bands are close to their baseline values (close to zero), indicating mostly uncorrelated fluctuations with a flat spectrum.

### 4.2. DFA Analysis

**Figure 4:**
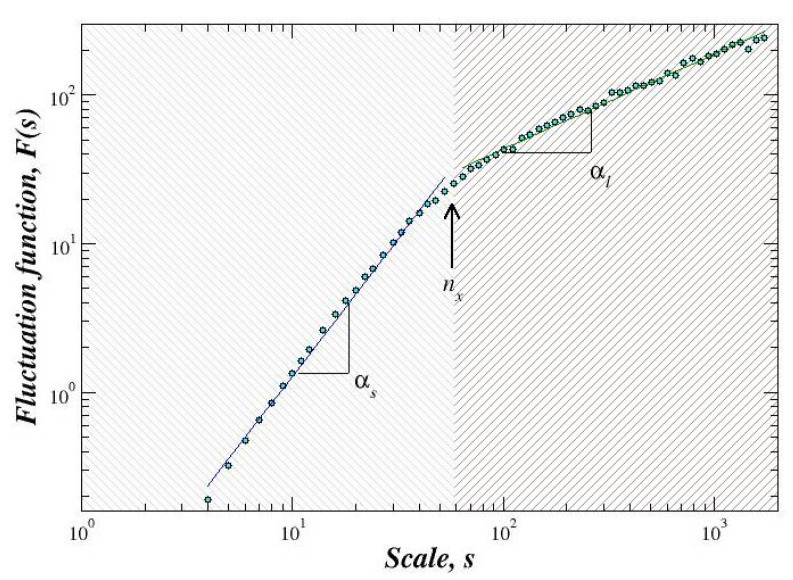
Fluctuation function F(s) of a representative segment of instantaneous amplitude corresponding to the frequency band $\Delta f_{1}$ during October 1994 in Acapulco station. A crossover $n_{x}$ is observed, which separates two regions. For short scales ($s<n_{x}$), $\alpha_{s}$ is close to 2, which corresponds to a very regular (tending to continuous functions) time series, while for large scales ($s>n_{x}$), $\alpha_{l}$ is close to one, i.e., close to 1/f noise.

To obtain further insights in the evaluation of the fluctuations observed in some periods of the records from the three stations and their relation with EQ occurrences, we apply the DFA method to segments of the same length as in the case of power spectral analysis but to data corresponding to instantaneous amplitudes and frequencies [30]. A representative case of the behavior of F(n) versus *n* for some periods during 1995 is shown in Figure 4. As we can see in this plot, two different scaling exponents can be defined for describing correlations, i.e., the crossover scale $n_{x}$ divides the F(s) function into two regions: Short scales ($s<n_{x}$) and large scales ($s>n_{x}$). For the estimation of the scaling exponents ($\alpha_{short}$ and $\alpha_{large}$) and the crossover point, we consider the procedure which consists in detecting the best two fits within two regions by minimizing the total fit errors [24]. We recall that the calculations of the DFA were done for filtered series according to the frequency-bands defined for the power spectrum analysis (see Section 4.1). For instantaneous frequency series, we found a crossover with two scaling α-exponents in all the frequency bands, however, no clear differences were detected when observing the whole time interval under study (data not shown).

**Figure 5:**
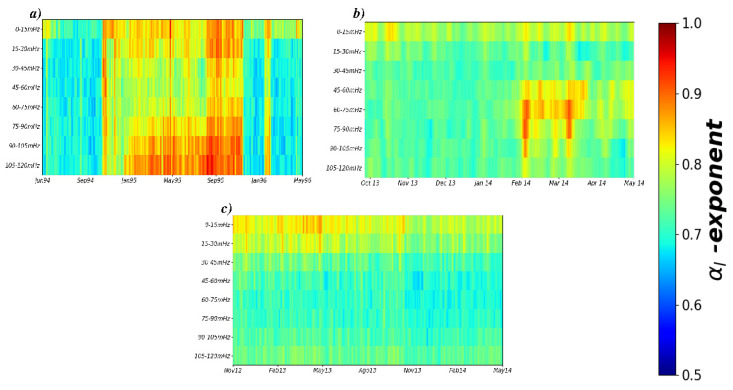
Heat map of evolution of the correlation $\alpha_{l}$ exponent for eight frequency bands obtained with data from three stations in the South Pacific Coast of Mexico located at (**a**) Acapulco, (**b**) Petatlán and (**c**) Pinotepa Nacional.

For amplitude data, we found noticeable differences between different periods of time and between frequency bands for Acapulco and Petatlán stations, especially for scaling exponents corresponding to large scales ($\alpha_{l}$). We notice that, for short scales, the values of $\alpha_{s}$ were always close to 2. In the following paragraphs, we only report the results of $\alpha_{l}$. Figure 5a shows the evolution of the $\alpha_{l}$ exponent for Acapulco station (see also [22]). For the period June 1994 until October 1994, we see that the correlation exponents fall within the interval [0.65,0.73], which are close to the uncorrelated noise ($\alpha_{l}=0.5$); from November 1994 until October 1995, the exponent values fall within the range [0.8,0.97], indicating that long-range correlations are present in almost all amplitude series; and for the period between November 1995 and May 1996, the $\alpha_{l}$ exponent values are quite similar to the first segment with values close to 0.5.

Results for the Petatlán station are shown in Figure 5b, where for the period from January 2014 until the end of March 2014 and for intermediate frequency bands, the correlation exponents are within the range [0.82,0.97]. This region is markedly different compared with the rest of the values in the plot, confirming that the identification of long-range correlated dynamics in the amplitudes occurred during a well-defined time interval a few weeks before the EQ occurrence.

The control station in Pinotepa Nacional leads to a more homogeneous distribution of exponents along the whole time interval and frequency bands, which mostly resemble uncorrelated fluctuations close to $\alpha_{l}=0.5$ (see Figure 5c).

## 5. Discussion and Conclusions

The two analyses presented here indicate that significant changes in geoelectric fluctuations occurred during the periods under study for Acapulco and Petatlán stations. These changes were expressed by means of higher values of power density, especially at low bands, and the appearance of correlated dynamics. The alterations followed clear transitions starting from uncorrelated noise to correlated dynamics and then uncorrelated fluctuations. Interestingly, the process of uprising of the power and correlated dynamics (expressed as $\alpha_{l}\approx 1$) in the signal from Acapulco station occurred approximately nine months before the main shock $M_{s}7.4$ EQ (with its epicenter ∼110 km away from the station), and a similar feature was observed for Petatlán data; however, for this case, the alterations appeared three and a half months before the $M_{s}7.2$ EQ (with its epicenter ∼57 km away from the station). The existence of the relaxation–EQ–preparation–main shock–relaxation process described in terms of power spectrum and DFA of amplitudes is in full agreement with previous analysis of geolectric data by means of multiscale entropy and correlation analyses [24,34]. We remark that the DFA of amplitudes (Figure 5) incorporates a qualitatively visual way to detect local correlated fluctuations, which can be used as a tool to discriminate and characterize a complex variation in noisy geoelectric time series. Our empirical study is based on recordings from only three stations; in order to have more conclusive characterizations, additional analyses are needed with a more robust database in the number of stations, signals and periods of observation. It is also necessary to develop theoretical models of EQ precursors. In summary, our results contribute to support for the identification of anomalies that are present in a variety of signals and behaviors, and which are likely related to the imminence of earthquakes M>7.
